# Improving access to medicines through centralised dispensing in the public sector: a case study of the Chronic Dispensing Unit in the Western Cape Province, South Africa

**DOI:** 10.1186/s12913-015-1164-x

**Published:** 2015-11-17

**Authors:** Bvudzai Priscilla Magadzire, Bruno Marchal, Kim Ward

**Affiliations:** University of the Western Cape, School of Public Health, Private Bag X17, Bellville, 7535 South Africa; Institute of Tropical Medicine, Antwerp, Belgium; University of the Western Cape, School of Pharmacy, Bellville, South Africa

**Keywords:** Centralised dispensing, Access to medicines, Medicines supply chain, Medicines distribution, Chronic diseases, Pharmaceutical services, Chronic Dispensing Unit, Missed appointments, Western Cape, South Africa

## Abstract

**Background:**

The Chronic Dispensing Unit (CDU) is an out-sourced, public sector centralised dispensing service that has been operational in the Western Cape Province in South Africa since 2005. The CDU dispenses medicines for stable patients with chronic conditions. The aim is to reduce pharmacists’ workload, reduce patient waiting times and decongest healthcare facilities. Our objectives are to describe the intervention’s scope, illustrate its interface with the health system and describe its processes and outcomes. Secondly, to quantify the magnitude of missed appointments by enrolled patients and to describe the implications thereof in order to inform a subsequent in-depth empirical study on the underlying causes.

**Methods:**

We adopted a case study design in order to elicit the programme theory underlying the CDU strategy. We consulted 15 senior and middle managers from the provincial Department of Health who were working closely with the intervention and the contractor using focus group discussions and key informant interviews. In addition, relevant literature, and policy and programme documents were reviewed and analysed.

**Results:**

We found that the CDU scope has significantly expanded over the last 10 years owing to technological advancements. As such, in early 2015, the CDU produced nearly 300,000 parcels monthly. Medicines supply, patient enrollment processes, healthcare professionals' compliance to legislation and policies, mechanisms for medicines distribution, management of non-collected medicines (emanating from patients’ missed appointments) and the array of actors involved are all central to the CDU’s functioning. Missed appointments by patients are a problem, affecting an estimated 8 %–12 % of patients each month. However, the causes have not been investigated thoroughly. Implications of missed appointments include a cost to government for services rendered by the contractor, potential losses due to expired medicines, additional workload for the contractor and healthcare facility staff and potential negative therapeutic outcomes for patients.

**Conclusions:**

The CDU demonstrates innovation in a context of overwhelming demand for dispensing medicines for chronic conditions. However, it is not a panacea to address access-to-medicines related challenges. A multi-level assessment that is currently underway will provide more insights on how existing challenges can be addressed.

**Electronic supplementary material:**

The online version of this article (doi:10.1186/s12913-015-1164-x) contains supplementary material, which is available to authorized users.

## Background

Access to essential medicines is considered a fundamental part of universal health coverage and a key element for the delivery of services and high-quality care [1]. A 2012 assessment of the South African health system underscored the need to give a higher priority status to medicines supply chains as they affect various dimensions of access to medicines and health care utilisation in general [2]. Although South Africa offers free primary health care (PHC) services in the public sector, subscribes to an essential medicines programme [3] and provides free medicines at PHC level [4], there are persistent challenges that hinder sustainable access to medicines. The increasing burden of disease [5], coupled with a general shortage and maldistribution of health professionals (private-public, urban–rural) [6], for example, threaten the ability of supply chain systems to function optimally. Shortages in all areas of pharmacy practice are common. Vacancy rates for pharmacists in the public sector of up to 76 %, were reported in one province and only 29 % of pharmacists were working in the public sector as of 2010 [7]. Various reforms and interventions have been implemented to address shortages of pharmacists, such as the introduction of incentives, the impact of which is yet to be determined on pharmaceutical human resource trends [8] and new models of centralised dispensing of medicines for chronic conditions [9, 10]. In this article, we report on a centralised dispensing intervention in the Western Cape Province, in South Africa.

### Western Cape Province’s response to strengthen access to medicines

Each of South Africa’s nine provinces has its own legislature, premier and executive council, and specific population and economical characteristics. The Western Cape is South Africa’s most cosmopolitan province, with a population of just under six million in 2011 [2]. The 2010 mortality profile reported that about 65 % of deaths occurred in the metropolitan district of Cape Town [11], which has the greatest proportion of patients and the greatest pressure on health services [9]. HIV/AIDS and non-communicable diseases (NCDs), account for a large proportion of premature mortality [12]. The healthcare system is two-tiered, consisting of a public and a private sector. However, the vast majority of the population (more than 75 %) is dependent on the public sector for, inter alia, supply of medicines [13].

The Western Cape Department of Health (WCDoH) established an out-sourced, centralised dispensing intervention known as the Chronic Dispensing Unit (CDU). Introduced in December of 2005, the CDU dispenses medicines for stable, public- sector patients with the aim to: reduce pharmacists’ workload (by relieving pharmacy staff from repetitive and time consuming tasks that detract from patient-focussed elements), decongest health facilities (hereafter referred as facilities) and improve the patient experience by reducing waiting times [9, 10]. The contractor, is responsible for specific supply chain functions which are elaborated on in later sections of this article.

The CDU was initially implemented as part of the province’s first strategic vision for health care (Health Care 2010), which acknowledged the necessity to substantially improve the quality of care of the health service, while recognising that “*One of the biggest challenges facing the Department is the need to ensure that its workforce meets the challenges of service delivery within a changing environment with a sizeable burden of disease.*” [14]. Since its establishment in 2005, the CDU has remained a significant part of the province’s plans. The current provincial strategy (Health Care 2030) states that “…*it is expected that the CDU will be well-established in future and will assist to address the increasing demand for efficient dispensing of chronic medicines, which are expected to form the bulk of the burden of disease in the next two decades*” [15]. In light of this, the CDU has been presented as part of the motivation for an increase in the health budget allocation [16], and a huge financial investment towards this service has been made. The current five-year contract between the government and the contractor, which commenced in 2012, is valued at 500 million South African rands [17], which was approximately 62,5 million United States dollars in 2012.

Despite the leadership’s commitment and efforts, several operational challenges exist. Among these challenges, the trend of missed appointments by patients is a concern to the WCDoH and our study was commissioned to investigate this issue. Within the context of this intervention, the term “non-collected medicines” is used to refer to pre-packed Patient Medicine Parcels (PMPs) that are not picked up by patients on or close to the scheduled date and are subsequently returned to the CDU. Monthly collection statistics are a key monitoring indicator of the intervention’s performance.

In this paper, we provide a comprehensive description of the operations of the CDU and seek to gain a better understanding of the current issue of concern—i.e. missed appointments. Earlier articles described the CDU in its initial stages of implementation focusing mostly on the dispensing processes [9, 10]. We set out to elicit the programme theory (defined as processes planned to achieve certain outcomes), which according to Van Belle et al. [18] is useful for understanding complex interventions. More specifically, this study aims to identify the actors’ interpretations of how the CDU’s activities are linked to the outcomes. Therefore, our objectives are: (1) to illustrate the CDU’s interface with the health system and describe its coverage, dispensing capacity and beneficiary profile; (2) to quantify the magnitude of missed appointments by patients and (3) to describe the implications thereof in order to inform a subsequent in-depth empirical study on the underlying causes.

## Methods

### Design

We adopted the case study design. This approach is appropriate because of the limited literature on the intervention and the need to understand how certain processes take place in order to comprehend the phenomenon [19].

Data were collected from multiple sources using focus group discussions, key informant interviews and document and literature reviews. We consulted 15 purposively selected key informants: senior and mid-level managers within the WCDoH involved in policy development and implementation of the intervention; and the current contracter (UTI Pharma).

The breakdown of respondents is provided in Table 1. Key informant perspectives were complemented by a review of published articles. We also carried out a review of CDU-related documents, including service level agreements, standard operating procedures, quarterly reports, conference proceedings, press statements, academic theses as well as routine data collected by the CDU. We developed a data collection tool that listed pre-determined variables of interest (e.g. coverage, dispensing capacity, demographics of beneficiaries and non-collected medicines). It also contained open- ended questions on processes and the issue of medicine non-collection. Data was collected between 2013 and 2014.

**Table 1:** Study participants

Level	Participant description	Number of participants	Research method used
Senior management	Western Cape Department of Health personnel	3	1 focus group discussion
Middle management	Sub-structure pharmacist managers	5	Key informant interviews (2 face-to-face and 3 telephonic)
Implementation team and support	Western Cape Department of Health personnel and the contractor	7	2 focus group discussions

We started the analysis of qualitative data by developing descriptive narrative accounts to map the intervention processes. We analysed the quantitative data using descriptive statistics in Microsoft Excel, including means and frequencies on age, gender, coverage, dispensing capacity and non-collected medicines. For data on non-collected medicines, we focused only on 2014 data, post introduction of the revised “returns policy” for healthcare facilities as data quality was expected to improve as a result of the policy revisions. For validation, we used member checking [20]. We circulated the draft manuscript to implementers of the intervention and invited comments.

Ethics approval for this study was granted by the Senate Research Committee at the University of the Western Cape (Ref: 11/7/8). Consent to interview and record interviews was obtained from participants. They were also informed of their right to withdraw from the interview at any time. To ensure anonymity, participants were assigned codes that were known only by the first author.

## Results

### Implementation context

The administration of public sector health services in the Western Cape Province falls under either of two jurisdictions. The provincial authority (WCDoH) administers a number of urban and rural facilities at the primary, secondary and tertiary levels of healthcare. The Cape Town Metropolitan municipality administers most primary healthcare clinics within its jurisdiction.

Healthcare facilities in this province rely on two complementary methods of medicines dispensing. Traditionally, patients with acute conditions and patients who are not yet stabilised on therapy for chronic conditions obtain their medicines at the dispensary of the healthcare facility that they use. On the other hand, the CDU is designed to dispense medicines for stable patients.

Key informants estimated that about 60 % of all patients with chronic conditions in the province obtained medicines through the CDU, although this is yet to be verified empirically by the WCDoH. Medicines dispensed by both the CDU and public-sector healthcare facilities are sourced from the government-owned Cape Medical Depot (CMD).

### Mapping the intervention processes

**Fig. 1:**
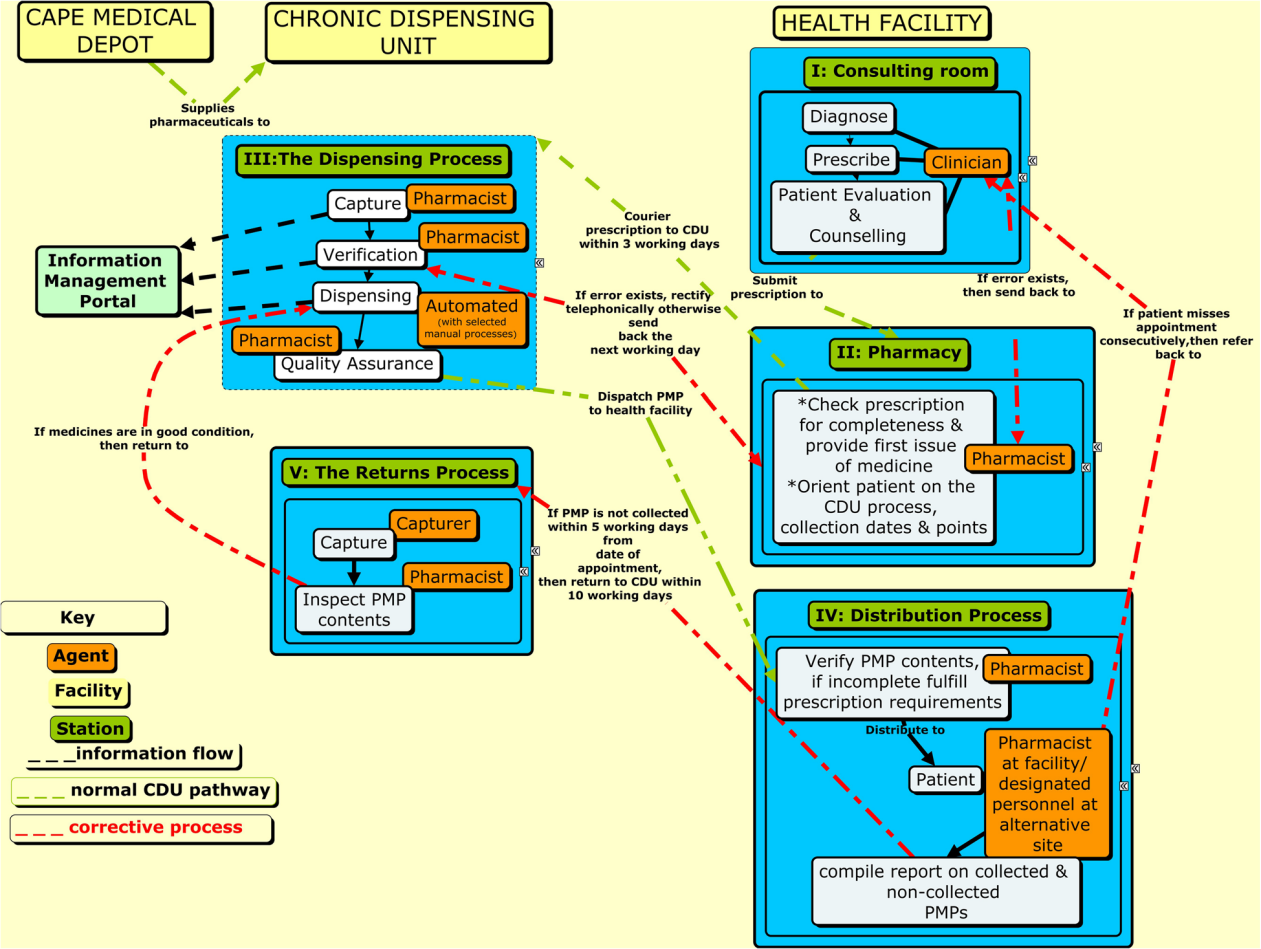
Mapping the process between the CDU and health facilities

We mapped the key processes between the CDU, the CMD and healthcare facilities, and identified the corresponding actors/stakeholders such as clinicians, the contractor and patients. Furthermore, we identified each actor’s responsibility and the relationships between the different actors (Fig. 1). Full narrative descriptions of the actors and processes are provided as Additional file 1. Although we depicted one healthcare facility as an example, this process is similar for all facilities registered with the CDU.

An implementation team, comprising WCDoH personnel and the contractor is at the core of the implementation strategy. This team orients new facilities to CDU policies in order to promote effective uptake of the intervention. Thereafter, implementation support is maintained by liaison officers appointed by the contractor to facilitate smooth roll-out of the intervention.

### Scope of the intervention

#### Coverage

The CDU has and continues to apply a phased approach to enrolling healthcare facilities. Using various WCDoH reports, we tracked the increase in this enrollment. The CDU commenced with eight urban healthcare facilities in 2005. By mid2008, just over 40 facilities were enrolled and later that year, the incorporation of rural regions started. By the end of 2013, over 100 facilities were enrolled, reaching 216 facilities in early 2015. The CDU more recently focused on enrollment of rural facilities and supporting decentralised pick-up points. Although PMPs are generally delivered to a healthcare facility, actual distribution is also occurring at alternative sites, such as mobile clinics, community clubs, old age homes and workplaces, most of which are linked to the nearest healthcare facilities. When healthcare facilities register alternative distribution sites with the CDU, PMPs are labelled seperately from those distributed from the healthcare facilities. The CDU had 2724 registered alternative sites at the end of 2014.

### Dispensing capacity

With an average of five to six items per prescription (which are all packaged in one PMP), the CDU dispensed over one million items each month in early 2015.

The first batch of PMPs in 2005 was 984 in total. This increased to almost 20,000 PMPs by the end of 2006 and almost 80,000 by the end of 2007. Over the next 4 years (up to 2011), 100 % growth occurred (80,000 to 160,000). Growth slowed down to 25 % between 2011 and 2013 (160,000 to 200,000 per month). This was most likely due to the change-over processes to a different contractor. Complexity of data transfer between the out-going and in-coming contractors partly affected the new contractor's ability to continue the service efficiently. This was further compounded by the implementation of new business processes and the commissioning of sophisticated dispensing equipment [21]. For the first few weeks of the transition, the new contractor and WCDoH jointly reverted to manual dispensing as an interim measure. WCDoH suspended some facilities from the CDU for about 3 months to allow the service to stabilise again.

Between 2013 and 2014, the dispensing capacity steadily increased with over 350,000 PMPs produced in October 2014. This high volume of PMPs was partially explained by the four months’ supply of antiretroviral therapy to accommodate the December/January festive period which is associated with patients travelling to their home provinces. The dispensing volume per month in the first quarter of 2015 was approximately 300,000 PMPs.

About 77 % of PMPs were delivered to urban healthcare facilities and 23 % to rural ones. The number of PMPs delivered to each facility ranged from under 1,000 to about 15,000 per site, per month. The increased dispensing capacity was facilitated by technological advancements, including largely automated processes for certain functions such as picking, packaging and labelling of medicines.

### Enrollment of patient beneficiaries

According to the procedure, patient enrollment should be based on a clinician’s assessment of the patient’s clinical stability. Patients who fail to achieve clinical stability and those with conditions demanding more regular monitoring (e.g. certain mental health conditions) or taking medicines requiring stricter control (e.g. benzodiazepines) should be excluded.

### Patient characteristics: age, gender and disease profile

**Fig. 2:**
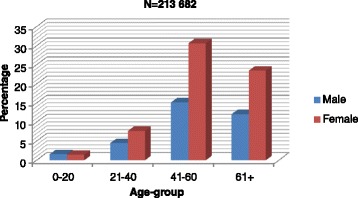
Distribution of CDU beneficiaries by age and gender

In early 2015, the CDU had 213,682 active patients (85 % urban and 15 % rural). Males constituted 34 % and females 66 % of the cohort. Slightly more than 80 % were over the age of 40 years, illustrating that the CDU served a predominantly adult population (Fig. 2).

It was not possible to evaluate the disease patterns from the CDU data since up to this point; the CDU does not capture patient diagnosis data. However, dispensed items were classified according to the Medi-Span Generic Product Identifier (GPI) classification. This is a hierarchical identifier, which provides specific information about medicines [22] and which may, to some extent, provide insights into patient diagnosis. We found that in 2013 and 2014 anti-hypertensives, diuretics, anti-diabetic agents, analgesics/antipyretics as well as bronchodilators constituted more than 50 % of the items dispensed each month. About 12 % of the PMPs contained HIV treatment.

### Missed appointments by CDU beneficiaries

At inception, a monthly allowance of 4 % "non-collection" was factored in to accommodate for loss-to-follow-up or death. However, we found that for the year 2014, an estimated 8 % to 12 % of PMPs were returned to the CDU as a result of non-collection. These were likely to be conservative estimates, since many facilities under-reported on collection statistics: the percentage of facilities that duly reported each month was only in the range of 24 % to 67 %. Furthermore, rural areas were excluded in the analysis since enrollment of most facilities was recent and the intervention had not stabilised. Some key informants suspected that since PMP collection statistics were regarded as a performance indicator, there was a disincentive to report statistics that could potentially be viewed negatively. In 2014, the WCDoH revised the “returns policy”, requiring healthcare facilities to strictly adhere to the returns time-frame i.e. within 10 working days from the scheduled collection date. Previously, some healthcare facilities kept non-collected PMPs for months to over a year before returning them to the CDU.

We found that missed appointments had several implications. First, medicines expired before they could be redistributed by the CDU. Second, the CDU’s average monthly consumption data were being distorted and subsequently impacted negatively on forecasting. Third, missed appointments imposed a financial burden on the government as the service provider is paid on a “fee-per-PMP-delivered” basis, meaning that every PMP that is not collected is still to be paid for yet it does not reach the patient. Fourth, missed appointments increased the contractor’s workload due to additional administrative processes and the efforts required to re-integrate stock into the dispensing system. At the healthcare facility level, missed appointments also generated more work for the pharmacy personnel, as they had to absorb medicines into the local pharmacy (if PMPs were not returned to the CDU). Furthermore, facility-based pharmacists dispensed medicines from the pharmacy or dispensary if the patient presented late and in some cases the clinician also had to consult with the defaulter patient. This undermined the efficiency benefit of the CDU. Finally, there were concerns of possible negative outcomes if patients missed appointments and subsequently defaulted on treatment.

We enquired about the possibility of identifying “best practice” facilities for benchmarking purposes. Key informants suggested that missed appointments were a wide-spread problem and that the variation between facilities was difficult to discern because of under-reporting, as alluded to earlier. However, respondents suggested that inclusion of urban healthcare facilities in benchmarking attempts was more ideal as the intervention has entered into the routine stage in these facilities. Cohort size was also perceived to influence patient management practises, i.e. larger patient cohorts were presumably more challenging to manage. One informant said, “*If you can solve it for these (large) sites, then you have solved it for the rest.*” There was a general interest to focus on improving collection of medicines for treating NCDs, since the disease burden was higher than that of HIV in this province and disease programmes were presumably much less developed for NCDs than for HIV.

## Discussion

This paper describes the gradual expansion of the CDU over the last 10 years. It also shows how missed appointments by patients are an important problem in CDU implementation [9] and that the causes of this have not been investigated thoroughly. In general, limited studies have been conducted on the CDU, there are no baseline data and a comprehensive evaluation is yet to be conducted. To prepare for further in-depth research, we carried out this exploratory case study, which provides a detailed description of the actors and components of the intervention. It points to some preliminary explanations about how the CDU is expected to work (the underlying assumptions, planned intervention and expected results). It also shows how the programme is actually running and it explored the actual results.

This study has some limitations. First, we are as of yet unable to present clear trends of missed appointments. Data quality was questionable due to under-reporting by healthcare facilities and conflicting data sources. In addition, data on patient diagnosis and outcomes which could have informed some aspects of our study have not been captured up to this point.

Despite these limitations, our preliminary results are in line with the (scant) publications. Missed appointments are not a new phenomenon to this intervention [9] or in healthcare provision in general [23–27]. If the same problem has persisted, what should be done differently to attain a different outcome? Some authors have suggested that unexpected results could be because a good intervention theory is not being carried out well or the problem is the theory itself [28]. In the case of the CDU, existing evidence suggests to some degree that the CDU objectives have been achieved and cite benefits such as reduced waiting times [9, 10, 29], patients’ improved experiences with healthcare services and their motivation to remain stable, increased time for patient counselling [9, 10], and pharmacists’ ability to serve more than double the number of people they served prior to CDU implementation [29]. Despite these reported benefits, however, we report  the difficulty to ascertain how most of the conclusions were reached, the sustainability of the gains and the inability to generalise the findings. There are also differing views which show that implementation results might be variable. For instance, Munyikwa’s study found that pharmacists’ workload had not decreased as anticipated. Instead, pressure shifted from dispensing to managerial and administrative tasks and pharmacists reported that the patient base had increased. As a result, time for  patient counselling was still limited. In addition, only a few patients reported reduced travelling costs [29]. This was not surprising given that the sample only consisted of patients who collected medicines from the healthcare facility and not from alternative sites in the community.

### Implications for further research

There is a dearth of literature on models of centralised dispensing. Also, out-sourcing of selected supply chain activities is considered to be  minimal in low-and-middle-income countries [30]. While we are aware that centralised dispensing occurs in the private sector in other countries, particularly high-income countries, this has not been documented in Africa. To our knowledge, the CDU is the first public sector, large-scale, centralised dispensing model in South Africa, and the only such model in Africa. As a result, implementing it without experiences from similar interventions to learn from was cited as a challenge [9]. However, despite this limited evidence, - centralised dispensing is gaining momentum in South Africa, especially as a part of the on-going National Health Insurance pilot programme in other South African provinces [31]. This underscores how centralised dispensing is a preferred strategy for improving access to medicines for public- sector patients in South Africa [32]. This paper advances the understanding of the CDU and lays a foundation for future work that aims to improve the intervention and provide lessons for similar models. This is crucial because the challenges that led to the establishment of the CDU are not unique to South Africa. Many countries with low economic indicators tend to have relatively similar challenges in their health systems [33].

The lack of evidence to explain possible causes for missed appointments call for in-depth research into CDU implementation. Placing the known implementation problem (in this case, missed appointments) as a starting point to enquiry has been cited as a useful way to understanding interventions [28]. It is likely that implementation results will be variable across healthcare facilities. Investigating facility-specific characteristics, such as human resources, infrastructure and staff motivation [34] and the impact of the intervention on the healthcare provider, patient access to treatment and difficulties in implementation could also be necessary [35].

## Conclusion

The CDU in the Western Cape province in South Africa reflects innovation in organisation, structure and delivery of healthcare in a middle- income country with a substantial demand for medicines for chronic conditions. Such a model has the potential to increase access to medicines in other settings. However, it is not a panacea for overcoming all challenges pertaining to access to medicines. This study informed a multi-level assessment that is currently underway to understand the problem of missed appointments within the context of implementation related factors.

## Additional file

Additional file 1:
**Process description of the Chronic Dispensing Unit (CDU) and health facilities.** (DOCX 15 kb)

## Abbreviations

CDU: Chronic Dispensing Unit
CMD: Cape Medical Depot
LMICs: Low-and-Middle Income Countries
NCDs: Non-Communicable Diseases
PMP: Patient Medicine Parcel
WCDoH: Western Cape Department of Health
