# Supplementary material for: Improving access to medicines through centralised dispensing in the public sector: a case study of the Chronic Dispensing Unit in the Western Cape Province, South Africa
Source: BMC Health Serv Res. 2015 Nov 17;15:513. doi: 10.1186/s12913-015-1164-x (PMC4650275; doi:10.1186/s12913-015-1164-x)
Supplement: Additional file 1: — Process description of the Chronic Dispensing Unit (CDU) and health facilities. (DOCX 15 kb) [file 12913_2015_1164_MOESM1_ESM.docx]

**Additional file 1: Process description of the Chronic Dispensing Unit (CDU) and health facilities**

*I: Primary site (healthcare facility)* – This is where the CDU process is initiated. The clinician selects stable patients (i.e. those who do not require regular contact with the clinician). The patient receives a prescription repeatable for up to six months and a six-month follow-up clinical appointment. Based on the clinician’s evaluation at follow-up after stage, the same process is repeated after review. If the patient requires constant monitoring, a CDU script will not be issued.

*II: Pharmacy/Dispensary (facility)* **–** The patient takes their folder to the dispensary for the first issue of medicine. New prescriptions are submitted to the pharmacist who checks them for compliance with legislation and policies. The first issue of medicine is dispensed at the pharmacy after which repeats (monthly for NCDs in urban areas, bi-monthly for antiretroviral therapy in urban areas and bi-monthly for all conditions in rural areas) are dispensed by the CDU.

*III: CDU* – The contractor collects prescriptions from the pharmacy at the healthcare facility. At the CDU, prescriptions are verified to ensure that they are legible and comply with legislation before they are manually entered into a database. If the prescription does not comply, depending on the nature of the error, an appointed person at the CDU will either attempt to resolve the error telephonically or send the prescription back to the healthcare facility. If the prescription is compliant, it is prompted for dispensing. The processes that follow are: dispensing, packaging, labelling and sorting according to healthcare facilities and collection dates. The package containing the prescribed medicines is referred to as the Patient Medicine Parcel (PMP). The PMPs are bar-coded and can be batch tracked from the point of supply into the hand of the patient. This facilitates product quality control and enables the department to initiate a recall if necessary.

*IV: Pharmacy* – PMPs are delivered to the healthcare facility three working days before the scheduled collection date. The delivery is accompanied by a manifest, which details patient information including the date of their next appointment. Also, the CDU indicates if there were medicines required on the prescription that were out of stock. The pharmacist at the healthcare facility will check the manifest and fulfill the prescription requirements using medicines available in the pharmacy. Distribution of PMPs is done by a range of personnel and at different delivery points. A pharmacist or Community Health Worker linked to a non-governmental organisation may collect medicines from the healthcare facility and deliver them to patients’ homes or other selected points in the community. In most cases, a CDU patient is able to bypass administrative processes by proceeding straight to the collection point.

*V: Management of non-collected PMPs* – Patients are given five working days grace period should they miss their scheduled appointment. Thereafter, PMPs can either be returned to the CDU within 10 working days from the date of collection or stock can be absorbed into the healthcare facility’s pharmacy. Whether the pharmacist opts for the first or second option, reports on non-collected PMPs should be submitted to the CDU. The service provider has a mechanism for reintegrating returned stock into the bulk stock. However, non-collected PMPs from alternative sites, fridge items and opened PMPs do not qualify for reintegration. If a patient misses appointments consecutively, the prescription should be stopped and the patient must consult the clinician for counselling and assessment.

VI: *Information Management* – As different activities take place at the CDU, data is captured and stored in a database. Selected variables are provided to the Western Cape Department of Health monthly for monitoring and evaluation purposes. This includes number of processed prescriptions (including those rejected), medicines dispensed, and out-of-stock and number of non-collected PMPs. Collection and/or statistics are dependent on healthcare facilities’ reporting). Information management is a crucial aspect for monitoring and evaluation, and also financial reporting, as the service provider is paid per PMP delivered.

**All activities are governed by the relevant dispensing legislation.*

***The term “Pharmacist” is used to represent a pharmacist or a pharmacist assistant depending on the facility.*
